# Comparative outcomes of Bernese periacetabular osteotomy in adolescents, young adults, and middle-aged adults: a minimum two-year follow-up

**DOI:** 10.1302/2633-1462.79.BJO-2025-0361.R1

**Published:** 2026-09-14

**Authors:** Yu-Hung Chen, Chia-Che Lee, Kuan-Wen Wu, Yu-Han Chen, Shu-Hsin Yao, Tsung-Lin Lee, Ken N. Kuo, Ting-Ming Wang

**Affiliations:** 1 Department of Orthopaedic Surgery, National Taiwan University Hospital, Taipei, Taiwan; 2 Graduate Institute of Biomedical Electronics and Bioinformatics, National Taiwan University, Taipei, Taiwan; 3 Department of Orthopaedic Surgery, Ditmanson Medical Foundation Chia-yi Christian Hospital, Chia-yi, Taiwan; 4 Department of Orthopaedic Surgery, Fu Jen Catholic University Hospital, New Taipei, Taiwan; 5 Cochrane Taiwan, Taipei Medical University, Taipei, Taiwan

**Keywords:** Bernese periacetabular osteotomy, Developmental dysplasia of the hip, Patient-reported outcomes, Joint preservation surgery, Hip osteoarthritis, Radiological outcomes, Middle-aged adults, delayed union, hips, osteoarthritis, dysplastic hips, osteotomy sites, osteotomies, periacetabular osteotomy, patient-reported outcomes, modified Harris Hip Score

## Abstract

**Aims:**

Bernese periacetabular osteotomy (PAO) is a well-established procedure for symptomatic hip dysplasia in adolescents and young adults. Its application in middle-aged patients remains debated due to concerns regarding complications and uncertain outcomes. This study evaluated the safety and effectiveness of PAO in middle-aged patients by comparing minimum two-year outcomes with those of younger cohorts.

**Methods:**

We conducted a retrospective cohort study of patients who underwent PAO between June 2015 and December 2021 with a minimum two-year follow-up. Patients with previous hip surgery or systemic conditions affecting outcomes were excluded. The final cohort comprised 27 hips in 16 adolescents (Group A, 11 to 19 years), 24 hips in 18 young adults (Group B, 20 to 39 years), and 17 hips in 14 middle-aged adults (Group C, 40 to 57 years). Radiological parameters, complications, and patient-reported outcomes (PROs), including visual analogue scale (VAS) pain scores, and modified Harris Hip Score (mHHS), were compared among groups.

**Results:**

All age groups demonstrated significant improvements in radiological parameters and PROs. Median mHHS improved from 84.7 (IQR 84.7 to 84.7) to 95.7 (IQR 95.7 to 100) in Group A, 81.4 (IQR 75.9 to 84.7) to 95.7 (95.7 to 100) in Group B, and 73.7 (66.0 to 75.9) to 95.7 (90.2 to 100) in Group C. No significant between-group differences in radiological parameters or PROs were observed at final follow-up. Complication rates were comparable across groups except for osteotomy site healing. The middle-aged group demonstrated longer osteotomy union times than the adolescent and young adult groups (median 13 months (IQR 10 to 14) vs six months (IQR 6 to 11) vs six months (IQR 5 to 7); (p < 0.001). Delayed union did not adversely affect functional outcomes or rehabilitation.

**Conclusion:**

Carefully selected middle-aged patients with hip dysplasia demonstrated favourable radiological and functional outcomes after PAO despite longer osteotomy union times, with complication rates comparable with younger cohorts. These findings support the safety and effectiveness of PAO in selected middle-aged patients with dysplasia-associated Tönnis grade I to II osteoarthritis.

Cite this article: *Bone Jt Open* 2026;7(9):1204–1214.

## Introduction

Hip dysplasia is present in 20% to 40% of patients with hip osteoarthritis.^1^ Elevated articular cartilage contact stress in dysplastic hips contributes to the development of secondary osteoarthritis.^2^ Bernese periacetabular osteotomy (PAO) is widely used to treat symptomatic hip dysplasia in adolescents and young adults;^3^ however, its role in middle-aged patients remains controversial due to concerns regarding higher complication rates and variable clinical outcomes.

Although total hip arthroplasty (THA) remains the primary surgical option for middle-aged patients with dysplastic osteoarthritis, PAO has emerged as a joint-preserving alternative. Previous studies have reported mixed results, with some demonstrating outcomes comparable with younger cohorts or THA in carefully selected middle-aged patients,^4,5^ whereas others reported suboptimal clinical outcomes, higher failure rates, and increased procedure-related complications with increasing age.^6,7^ Major complication rates following PAO range from 6% to 37%,^8^ whereas complications in middle-aged patients remain less well-defined and are generally considered higher due to reduced healing potential and the greater prevalence of osteoarthritis.

Given the limited evidence regarding PAO in middle-aged populations, this study aimed to determine whether middle-aged PAO patients could achieve safety and efficacy profiles comparable with those of its well-established candidates: adolescents and young adults. We reviewed consecutive cases at our institution to compare: 1) radiological improvement; 2) patient-reported outcomes (PROs); and 3) complication profiles across different age groups.

## Methods

Between June 2015 and December 2021, 96 consecutive patients (123 hips) with hip dysplasia underwent PAO at the National Taiwan University Hospital. Patients were excluded if they had previous ipsilateral hip surgery altering acetabular orientation (21 patients, 21 hips), concurrent femoral procedures (12 patients, 15 hips), pelvic pathology associated with severe deformity or impaired bone healing (four patients, six hips), systemic conditions affecting baseline function or adherence to rehabilitation (six patients, eight hips), or less than two years of follow-up (five patients, five hips). All excluded cases were from the adolescent or young adult groups.

**Fig. 1:**
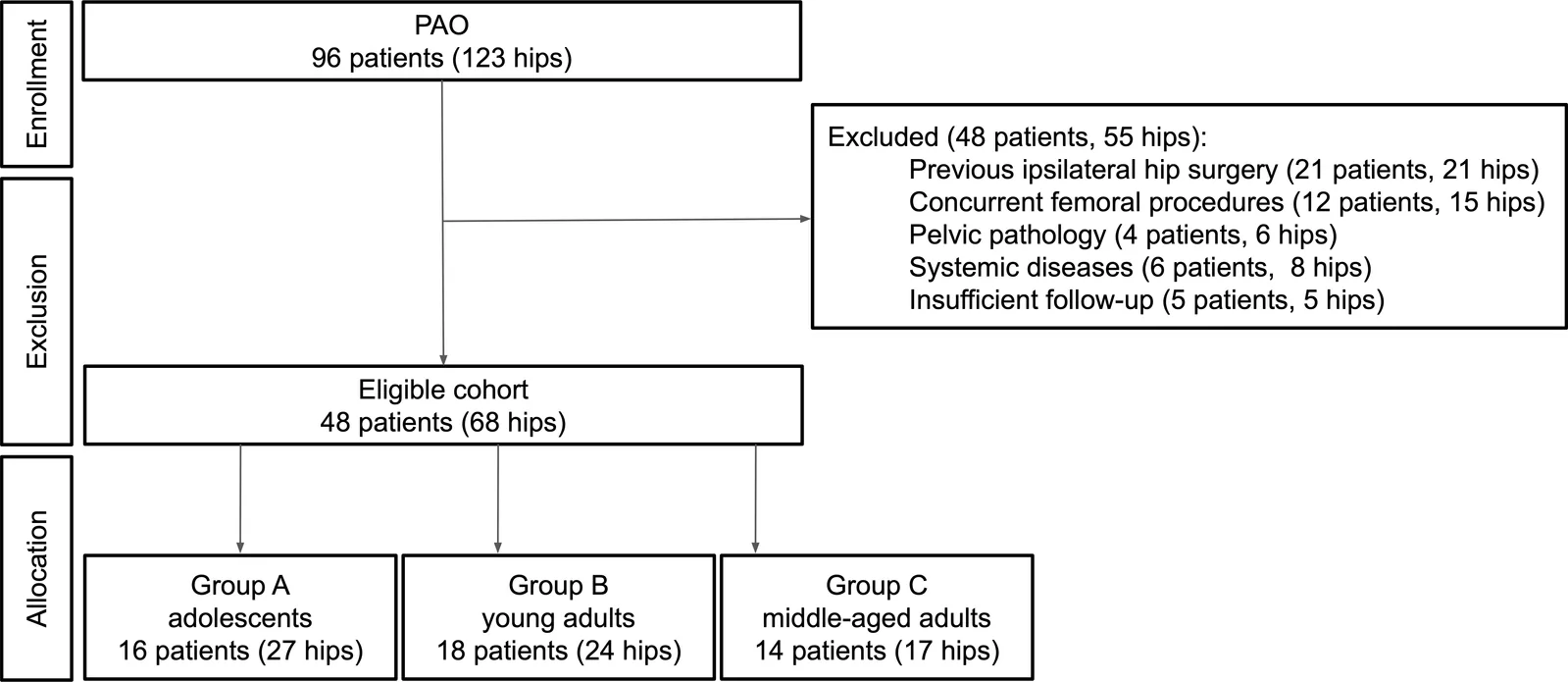
Flow diagram of patient inclusion. Group A comprised patients aged 11 to 19 years, Group B 20 to 39 years, and Group C 40 to 57 years. PAO, periacetabular osteotomy.

The final cohort comprised 48 patients (68 hips), stratified by age into three groups: adolescents (Group A, 16 patients, 27 hips; aged 11 to 19 years); young adults (Group B, 18 patients, 24 hips; aged 20 to 39 years); and middle-aged adults (Group C, 14 patients, 17 hips; aged 40 to 57 years (Figure 1). The median follow-up duration was 46.5 months (IQR 33.5 to 63.5). Preoperative Tönnis grading demonstrated milder osteoarthritis in Group A than in Groups B and C, with grade I/II distributions of 23/4, 15/9, and 9/8, respectively (Table I).^9^

**Table I.** Demographic details of patients who underwent periacetabular osteotomy in different age groups.

Variable	Group A	Group B	Group C	p-value			
				Across groups	A vs B	A vs C	B vs C
Patients, n (hips)	16 (27)	18 (24)	14 (17)				
Median age, yrs (IQR)	16 (14 to 17)	33 (26 to 36)	50 (44 to 52)				
Median BMI, kg/m^2^ (IQR)	21.1 (18.1 to 22.0)	23.7 (22.1 to 25.4)	23.8 (22.6 to 30.2)	< 0.001*	0.001†	< 0.001†	0.272†
**Sex, n (%)**				0.051‡			
Male	7 (25.9)	3 (12.5)	0 (0)				
Female	20 (74.1)	21 (87.5)	17 (100)				
**Side, n (%)**				0.889§			
Left	13 (48.1)	10 (41.7)	8 (47.1)				
Right	14 (51.9)	14 (58.3)	9 (52.9)				
**Tönnis grade for OA, n (%)**				0.042*	0.035†	0.021†	0.747†
Grade 0	0	0	0				
Grade I	23 (85.2)	15 (62.5)	9 (52.9)				
Grade II	4 (14.8)	9 (37.5)	8 (47.1)				
Grade III	0	0	0				

* Kruskal–Wallis test.

† Mann–Whitney U test.

‡ Fisher’s exact test.

§ Chi-squared test.

¶ Analysis of variance test.

OA, osteoarthritis.

### Surgical procedure

PAO was performed at our institution as originally described by Ganz et al^10^ using a modified Smith–Petersen approach with anterior superior iliac spine (ASIS) osteotomy. The fascia over the tensor fasciae latae was incised laterally to protect the lateral femoral cutaneous nerve. Sequential osteotomies of the ischium, superior pubic ramus, ilium, and posterior column were performed. The periacetabular fragment was mobilized with Schanz screws (DePuy Synthes, USA) and reoriented under direct visualization and fluoroscopic guidance, targeting a lateral centre-edge angle (LCEA) of 25° to 40°, an acetabular head index (AHI) > 80%, and restoration of physiological acetabular anteversion without retroversion or crossover sign.^11^

Following provisional fixation with Kirschner wires, hip range of motion (ROM) was dynamically assessed to ensure > 105° of flexion and > 20° of internal rotation at 90° of flexion without impingement. Definitive fixation was achieved using three screws (headless compression or cannulated, 6.5 to 7.0 mm). The ASIS was reduced and fixed, and the wound was closed over a closed-suction drain. Postoperatively, patients were restricted to toe-touch weightbearing for one month, followed by progression to full weightbearing with crutch assistance.

### Study outcomes

**Fig. 2:**
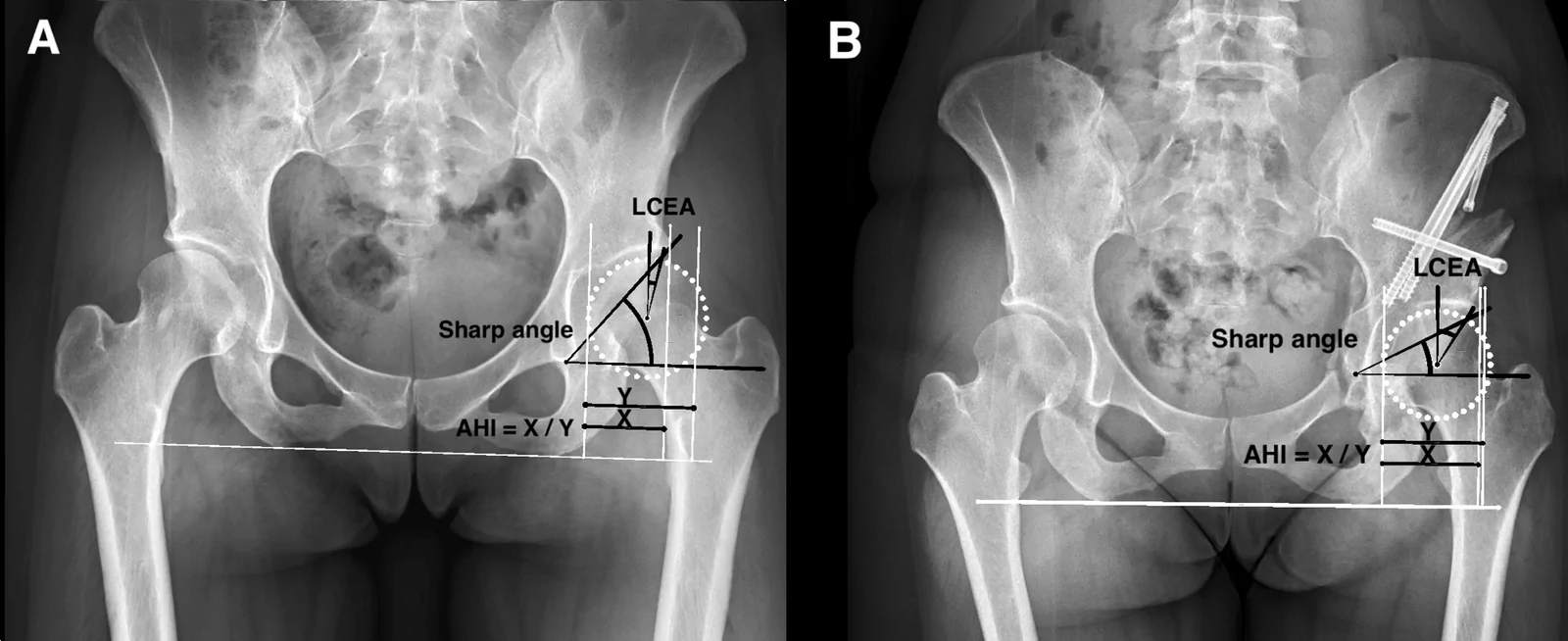
Measurement of the lateral centre-edge angle (LCEA), Sharp’s angle, and acetabular head index (AHI) on a) preoperative and b) Six-month postoperative radiographs from a 33 year-old female. A line connecting the ischial tuberosities was used as the horizontal reference for all measurements. The LCEA is defined as the angle between a vertical line perpendicular to the horizontal reference passing through the centre of the femoral head and a line extending from the femoral head centre to the lateral edge of the sourcil. The Sharp’s angle is defined as the angle between the horizontal reference and a line connecting the inferior aspect of the acetabular teardrop to the lateral edge of the acetabular roof. The AHI is defined as the ratio of the horizontal distance from the medial margin of the femoral head to the lateral edge of the acetabulum to the total horizontal diameter of the femoral head.

Radiological evaluations were performed preoperatively, immediately postoperatively, and at three, six, 12, and 24 months postoperatively, with extended follow-up for delayed union or nonunion. All measurements were obtained from weightbearing anteroposterior pelvic radiographs. To minimize ambiguity related to osteotomies near the acetabular teardrop, a line connecting the ischial tuberosities was used as the horizontal reference for both preoperative and postoperative measurements, which has been shown to provide equivalent LCEA measurements to the inter-teardrop line.^12^Assessed parameters included the LCEA,^12^ Sharp’s angle,^13^ AHI,^14^ and Tönnis grade for osteoarthritis (Figure 2). Intraobserver reliability was assessed at a one-month interval, and interobserver reliability was evaluated by an independent blinded observer (YHuC, YHaC). Interobserver reliability for Tönnis grading was good (κ = 0.718; p < 0.001, Cohen's kappa), as was intraobserver reliability (κ = 0.654 to 0.776; p < 0.001, Cohen’s kappa). Intra- and interobserver reliability for LCEA, Sharp’s angle, and AHI were excellent, with intraclass correlation coefficients ranging from 0.783 to 0.909.

PROs, including the visual analogue scale (VAS) for pain and the modified Harris Hip Score (mHHS) for pain and function,^15-17^ were recorded preoperatively and at a minimum two-year follow-up. The mHHS, derived from the original Harris Hip Score to include only pain and function domains (maximum raw score, 91 points),^16^ was normalized to a 0 to 100 scale using a multiplier of 1.1 to facilitate comparison with the original Harris Hip Score.^17^ Assessments were conducted via telephone interviews during the COVID-19 pandemic.

Complications included osteonecrosis, infection, nerve injury, decreased ROM, delayed union, nonunion, hardware irritation, and post-PAO pubic/ischial stress fracture. Delayed union was defined as a persistent radiolucent line across any osteotomy site at one year postoperatively,^18,19^ whereas nonunion was defined as persistent discontinuity at two years postoperatively. Discontinuity was defined as absence of cortical bridging without evidence of progressive interval healing on serial radiographs.^20^

### Statistical analysis

Normality was assessed using the Shapiro–Wilk test and quantile–quantile (Q–Q) plots. Normally distributed data were compared using one-way analysis of variance with Tukey’s honest significant difference test for post hoc analysis, and non-parametric data were analyzed using a Kruskal–Wallis test with a Mann–Whitney U test for post hoc comparisons. Pre- and postoperative outcomes within groups were compared using the Wilcoxon signed-rank test for non-normally distributed data and paired t-test for normally distributed data. Categorical variables were analyzed using a chi-squared test or Fisher’s exact test. Statistical significance was set at p < 0.05. Analyses were performed using Python v. 3.8 (Python Software Foundation, USA) with SciPy (SciPy Developers, USA) v.1.12.0.

## Results

### Radiological outcomes

At the two-year follow-up, all groups demonstrated significant improvements in radiological parameters. Mean LCEA increased in Groups A and B (both p < 0.001, paired *t*-test), and in Group C (p < 0.001, Wilcoxon signed-rank test), whereas the mean Sharp’s angle decreased in all groups (p < 0.001, all paired *t*-test). Median AHI also improved significantly in all groups (all p < 0.001, Group A and Group B: paired *t*-test; Group C: Wilcoxon signed-rank test). No significant between-group differences were observed in radiological parameters preoperatively or at final follow-up, indicating comparable acetabular correction across age groups (Table II).

**Table II.** Radiological outcomes of patients who underwent periacetabular osteotomy in different age groups.

Variable	Group A	Group B	Group C	p-value
Patients (hips), n	16 (27)	18 (24)	14 (17)	
**Mean LCEA, ° (SD)**				
Preoperative	15.2 (8.3)	13.9 (9.4)	16.1 (10.0)	0.726*
Postoperative†	42.4 (6.8)	43.5 (6.0)	45.2 (10.7)	0.500*
3 mths postop	41.1 (6.7)	41.6 (5.9)	43.9 (11.7)	0.528*
6 mths postop	40.9 (6.9)	41.3 (6.2)	44.3 (13.4)	0.418*
1 yr postop	40.1 (6.9)	41.1 (6.4)	43.8 (13.2)	0.385*
2 yrs postop	39.2 (6.7)	40.9 (6.9)	44.2 (13.0)	0.277*
**Mean Sharp’s angle, ° (SD)**				
Preoperative	48.6 (4.7)	49.3 (3.9)	49.5 (4.0)	0.733*
Postoperative†	26.0 (5.3)	26.4 (7.2)	25.6 (6.1)	0.923*
3 mths postop	27.5 (5.2)	27.3 (6.5)	26.5 (6.7)	0.865*
6 mths postop	27.9 (4.7)	28.0 (6.6)	26.5 (6.9)	0.707*
1 yr postop	28.0 (4.7)	28.6 (6.7)	27.1 (6.4)	0.742*
2 yrs postop	29.6 (4.0)	29.0 (6.0)	27.5 (5.9)	0.500*
**Median AHI, % (IQR)**				
Preoperative	70 (61 to 74)	65 (54 to 71)	64 (62 to 74)	0.567‡
Postoperative†	95 (90 to 100)	100 (91 to 100)	99 (86 to 100)	0.487‡
3 mths postop	91 (89 to 99)	96 (86 to 100)	96 (85 to 100)	0.677‡
6 mths postop	91 (86 to 99)	93 (86 to 100)	95 (86 to 100)	0.727‡
1 yr postop	90 (86 to 99)	90 (85 to 100)	95 (85 to 100)	0.736‡
2 yrs postop	91 (87 to 98)	98 (89 to 100)	95 (88 to 100)	0.442‡

* Analysis of variance.

† Taken within one week postoperatively.

‡ Kruskal–Wallis test.

AHI, acetabular head index; LCEA, lateral centre-edge angle.

### Patient-reported outcomes

Pain severity, assessed using the VAS, improved significantly in all groups, with median scores decreasing from 3 (IQR 2 to 3.5) to 0 (IQR 0 to 0) at final follow-up (all p < 0.001, Wilcoxon signed-rank test). Although Group C had higher preoperative VAS scores than Group A (p = 0.014, Mann–Whitney U test), postoperative VAS scores did not differ significantly among groups (p = 0.104, Kruskal–Wallis test).

Functional outcomes, assessed by mHHS, also improved significantly across all groups ( Group A and Group C p < 0.001; Group B p = 0.002; all Wilcoxon signed-rank test). Median mHHS increased from 84.7 (IQR 84.7 to 84.7) to 95.7 (IQR 95.7 to 100) in Group A, 81.4 (IQR 75.9 to 84.7) to 95.7 (IQR 95.7 to 100) in Group B, and 73.7 (IQR 66.0 to 75.9) to 95.7 (IQR 90.2 to 100) in Group C. Although preoperative mHHS differed significantly among groups, with the lowest scores observed in group C (p < 0.001, Kruskal–Wallis test, with the Mann–Whitney U test applied for post-hoc pairwise comparisons), no significant between-group differences were observed at final follow-up. Notably, the middle-aged cohort demonstrated the most substantial improvement from its lowest score preoperatively (Table III).

**Table III.** Patient-reported outcomes of patients who underwent periacetabular osteotomy in different age groups.

Variable	Group A	Group B	Group C	p-value			
				Across groups	A vs B	A vs C	B vs C
Patients, n	16 (27)	18 (24)	14 (17)				
**Median VAS (out of 10) (IQR)**							
Preoperative	3 (1 to 3)	3 (2 to 3)	3 (3 to 5)	0.028*	0.138†	0.014†	0.129†
Postoperative‡	0 (0 to 0)	0 (0 to 0)	0 (0 to 1)	0.104*			
p-value within group	< 0.001§	< 0.001§	< 0.001§				
**Median mHHS (out of 100) (IQR)**							
Preoperative	84.7 (84.7 to 84.7)	81.4 (75.9 to 84.7)	73.7 (66.0 to 75.9)	< 0.001*	< 0.001†	< 0.001†	0.009†
Postoperative‡	95.7 (95.7 to 100)	95.7 (95.7 to 100)	95.7 (90.2 to 100)	0.543*			
p-value within group	< 0.001§	0.002§	< 0.001§				

* Kruskal–Wallis test.

† Mann–Whitney U test.

‡ Taken at last follow-up, a minimum of two years postoperatively.

§ Wilcoxon signed-rank test.

mHHS, modified Harris Hip Score; VAS, visual analogue scale.

### Complications

**Fig. 3:**
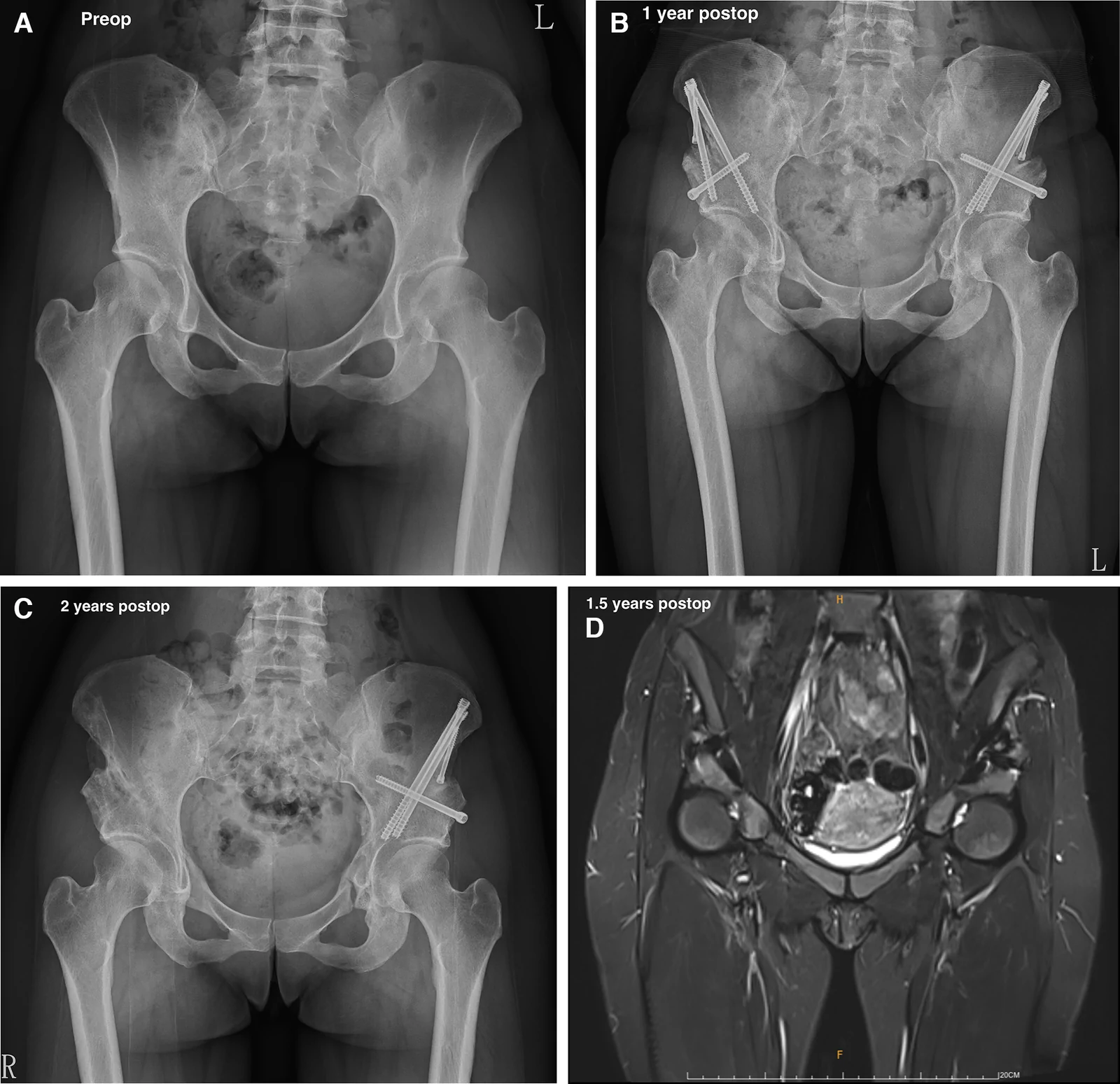
Sequential imaging of a 33-year-old female with bilateral symptomatic hip dysplasia who underwent staged bilateral periacetabular osteotomy (PAO). a) Preoperative weightbearing anteroposterior pelvic radiograph demonstrated bilateral hip dysplasia with lateral centre-edge angle (LCEA) values of 15.7° (left) and 19.5° (right). b) The patient underwent left PAO followed by right PAO five months later. One-year postoperative radiograph demonstrated improved femoral head coverage with LCEA values of 37.5° (left) and 36.4° (right), as well as delayed union of the left superior pubic ramus osteotomy site. c) Two-year postoperative radiograph demonstrated persistent discontinuity of the left superior pubic ramus osteotomy site, consistent with nonunion, with minimal interval change. d) T2 short tau inversion recovery MRI obtained 1.5 years postoperatively confirmed absence of cortical bridging at the left superior pubic ramus osteotomy site.

Comparative analysis demonstrated significant between-group differences in osteotomy site healing, whereas other complication rates were comparable (Table IV). The middle-aged cohort (Group C) had a significantly higher incidence of delayed union at one year (p < 0.001, chi-squared test), with odds ratios of 11.4 (95% CI 2.4 to 53.3) and 15.7 (95% CI 2.8 to 90.0) compared with the adolescent and young adult groups, respectively. However, delayed union was clinically asymptomatic and did not adversely affect rehabilitation or daily activities. At two years postoperatively, nonunion rates did not differ significantly among groups (p = 0.598, Fisher’s exact test). Seven osteotomy site nonunions were identified, including five pubic (Figure 3), one ischial, and one combined pubic and ischial nonunion. All nonunions were either asymptomatic or associated with mild local tenderness and did not require surgical intervention. Time to union also differed significantly among groups (p < 0.001, Kruskal–Wallis test), with the middle-aged group demonstrating a longer union time than the adolescent and young adult groups 13 months (IQR 10 to 14) vs six months(IQR 6 to 11) vs six months (IQR 5 to 7); (p < 0.001, Kruskal–Wallis test).

**Table IV.** Short-term complications of patients who underwent Bernese periacetabular osteotomy (PAO) in different age groups.

Variable	Group A	Group B	Group C	p-value across groups			
				Across groups	A vs B	A vs C	B vs C
Patients, n (hips)	16 (27)	18 (24)	14 (17)				
Further surgical intervention, n (%)	2 (7.41)*	1 (4.17)†	1 (5.88)‡	> 0.999§			
ONFH, n (%)	2 (7.41)	0 (0)	0 (0)	0.335§			
Wound infection, n (%)	0 (0)	1 (4.17)	0 (0)	0.603§			
Prolonged lateral thigh numbness (> 6 mths), n (%)	1 (3.70)	2 (8.33)	2 (11.8)	0.618§			
Sciatic nerve injury, n (%)	0 (0)	0 (0)	0 (0)	N/A			
Decreased ROM, n (%)	0 (0)	1 (4.17)	0 (0)	0.603§			
Hardware irritation, n (%)	1 (3.70)	0 (0)	0 (0)	> 0.999§			
Post-PAO pubic/ischial stress fracture, n (%)	3 (11.1)	0 (0)	2 (11.8)	0.203§			
**Discontinuity of osteotomy site**							
1 yr postoperative, n (%)	3 (11.1)	2 (8.3)	10 (58.8)	< 0.001¶	> 0.999§	0.002§	0.001§
2 yrs postoperative, n (%)	2 (7.41)	2 (8.33)	3 (17.6)	0.598§			
Median osteotomy union time, mths (IQR)	6 (5 to 7)	6 (6 to 11)	13 (10 to 14)	< 0.001**	0.036††	< 0.001††	< 0.001††

* Core decompression.

† Conversion surgery to THA.

‡ Revision surgery.

§ Fisher’s exact test.

¶ Chi-squared test.

** Kruskal–Wallis test.

†† Mann–Whitney U test.

N/A, not applicable; ONFH, osteonecrosis of femoral head; ROM, range of motion; THA, total hip arthroplasty.

No significant between-group differences were observed in other complications or reoperation rates. In Group A, two patients developed osteonecrosis of the femoral head requiring core decompression; one progressed from Ficat stage II to III, whereas the other developed newly diagnosed Ficat stage II disease with persistent pain.^21^ In Group B, one patient with preoperative Tönnis grade II osteoarthritis underwent conversion to THA at 20 months because of progression to Tönnis grade III osteoarthritis and worsening symptoms despite initial improvement. In Group C, one patient required early revision for excessive acetabular retroversion with mild subluxation.

**Fig. 4:**
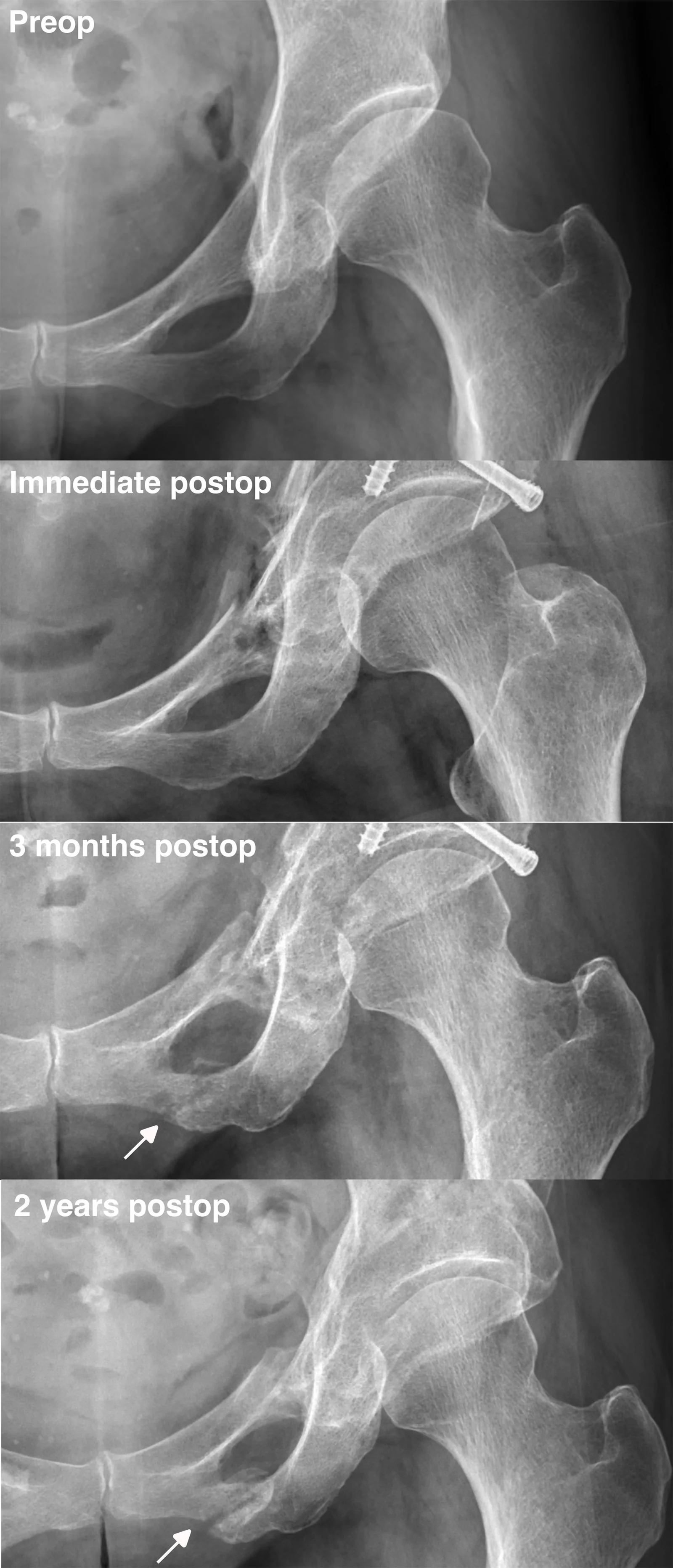
Serial radiological images of a 53-year-old female who underwent left PAO for symptomatic hip dysplasia. A left inferior pubic ramus stress fracture was first identified three months postoperatively (white arrow), with progressive surrounding callus formation observed on the two-year postoperative radiograph.

Additional complications included one superficial wound infection in group B (p = 0.603, Fisher’s exact test), prolonged lateral thigh numbness in five patients (p = 0.618, Fisher’s exact test), and pubic/ischial stress fractures in five patients (p = 0.203, Fisher’s exact test) (Table IV). One patient in Group A with combined ischial and inferior pubic ramus stress fractures remained asymptomatic despite persistent radiological nonunion. Four patients developed isolated inferior pubic ramus stress fractures (Group A, n = 2; Group C, n = 2), all of which demonstrated progressive surrounding callus formation without functional sequelae (Figure 4).

## Discussion

Age-related bone quality is a concern in middle-aged patients undergoing PAO, particularly regarding the ability to achieve and maintain adequate radiological correction. In the present study, sustained correction of the LCEA, Sharp’s angle, and AHI was observed across all age groups, with middle-aged patients demonstrating radiological outcomes comparable with younger cohorts throughout the two-year follow-up period. No evidence of loss of correction attributable to compromised bone quality was observed, likely owing to adequate osseous contact and stable internal fixation. These findings are consistent with previous studies reporting comparable radiological correction in middle-aged patients undergoing PAO. Teratani et al^22^ found no significant differences in postoperative radiological parameters between patients aged ≥ 50 years and younger cohorts following curved PAO. Similarly, Yilmaz et al^23^ reported comparable radiological outcomes between patients above and below 35 years of age after Bernese PAO.

The primary objective of PAO is to improve femoral head coverage through acetabular reorientation, thereby optimizing hip biomechanics and delaying osteoarthritic progression. Although sustained radiological correction was achieved across all age groups in the present study, the mean postoperative LCEA reached 41.3°, slightly exceeding the commonly accepted target range of 25° to 40°.^11^ Both under-correction and over-correction may compromise long-term outcomes, and postoperative LCEA values < 30° or > 40° have been associated with increased risk of conversion to THA.^24^ While under-correction may result in residual dysplasia, over-correction may lead to iatrogenic pincer-type femoroacetabular impingement (FAI), a recognized risk factor for persistent pain and progressive osteoarthritis.^25^ At our institution, FAI was routinely assessed intraoperatively after provisional fixation, targeting > 105° of hip flexion and > 20° of internal rotation at 90° of flexion without impingement. Temporary fixation was revised when impingement was detected, which may explain the favourable PROs despite relatively high postoperative LCEA values. Recent finite element analysis further suggests that a LCEA of 30° to 35° with approximately 15° of anterior rotation may represent an optimal biomechanical target in dysplastic hips.^26^ Collectively, these findings emphasize that the goal of PAO is to optimize femoral head coverage while avoiding over-correction and FAI.

The influence of age on PROs following PAO remains debated. In the present study, VAS and mHHS improved significantly across all age groups. Although postoperative VAS and mHHS at the two-year follow-up were comparable among groups, the middle-aged cohort had significantly lower preoperative mHHS, likely reflecting higher BMI and more advanced osteoarthritis. Nevertheless, this group demonstrated the greatest improvement in PROs. These findings are consistent with the prospective multicentre study by Muffly et al,^27^ which reported greater magnitude of improvement in patients aged ≥ 40 years despite comparable early postoperative outcomes across age groups. However, longer-term studies suggest gradual deterioration in older patients over time. Ito et al^28^ reported lower long-term HHSs and survivorship in older patients despite comparable five-year outcomes. A recent study with a mean 19.5-year follow-up identified higher preoperative Tönnis grade and BMI, rather than age alone, as major predictors of inferior long-term PRO measures.^29^ Collectively, these findings suggest that PAO candidacy should be determined by biological age, preoperative joint condition, and BMI rather than chronological age alone.^30^

Previous studies have reported preoperative mHHS values ranging from 55 to 67 points, improving to 85 to 92 points at mid- to long-term follow-up.^31-33^ Although the median postoperative mHHS in our cohort (95.7) was slightly higher than previously reported, baseline mHHS values were also comparatively high across all age groups (73.7, 81.4, and 84.7, respectively). The mHHS is known to have a ceiling effect, particularly in higher-functioning populations undergoing PAO, which may limit sensitivity for detecting subtle postoperative improvement.^34^ Furthermore, the mHHS has not been formally cross-culturally validated in Taiwanese or Mandarin-speaking populations. These limitations should be considered when interpreting the present findings.

A meta-analysis of 29 cohort studies reported PAO-associated major and minor complication rates of 4.3% and 14%, respectively, with peroneal nerve dysfunction, acetabular necrosis, and delayed union/nonunion being the most common major complications.^35^ Increasing age has been associated with higher complication rates following combined hip arthroscopy with PAO procedures,^36^ whereas younger patients (< 20 years) appear to have a lower risk of complications.^37^ In the present study, except for osteotomy site healing-related parameters, complication rates were comparable across age groups, including rates of reoperation, osteonecrosis of the femoral head, prolonged lateral thigh numbness, decreased ROM, hardware irritation, and pubic/ischial stress fractures.

Nonunion of osteotomy sites is a recognized complication following PAO, and advanced age has been identified as an independent risk factor for delayed union or nonunion at one year postoperatively.^19,20^ Consistent with previous reports, the middle-aged group in our study demonstrated a significantly higher rate of delayed union at 12 months (58.8%; 10 out of 17), whereas rates in the adolescent (11.1%; 3 out of 27) and young adult groups (8.3%; 2 out of 24) were comparable with previously reported rates of 8% to 17%.^19,20,38^ Kinoshita et al^18^ further demonstrated that discontinuity at the pubic osteotomy site decreased from 16.5% at one year to 5.2% at final follow-up, suggesting that radiological discontinuity at one year may represent delayed union rather than true nonunion. Similarly, substantial interval healing was observed in our middle-aged cohort between one and two years postoperatively, with persistent nonunion remaining in only 17.6% (3 out of 17) at two years and no significant between-group difference in nonunion rates (p = 0.598). All cases of delayed union and nonunion in our cohort were asymptomatic or associated with only mild local tenderness and did not require surgical intervention. Previous studies have reported inconsistent findings regarding the clinical impact of delayed union. One study demonstrated worse pain scores in patients with delayed union of the superior pubic ramus at one year postoperatively,^38^ whereas others found no significant differences in PROs between union and nonunion groups.^18,19^ Taken together with our findings, these data suggest that osteotomy site healing may continue beyond one year postoperatively, particularly in middle-aged patients. Accordingly, in the absence of significant symptoms, instability, or new-onset stress fractures, surgical intervention for delayed union may reasonably be deferred.

Our study had several limitations. First, the sample size was relatively small; however, consecutive cases from June 2015 to December 2021 were analyzed to reduce selection bias. Second, mHHS assessments were conducted via telephone interviews during the COVID-19 pandemic. Nevertheless, Sharma et al^39^ demonstrated good reliability of telephone-administered mHHS questionnaires compared with in-person evaluations in the context of THA follow-up, supporting the validity of our telephone-based data collection. Third, the relatively small cohort restricted sub-group analysis and reduced the power to detect rare complications.

In conclusion, this study provides additional evidence supporting the application of PAO in appropriately selected middle-aged patients. Although osseous consolidation at the osteotomy sites requires a longer period in this population, our findings suggest that middle-aged patients with hip dysplasia and Tönnis grade I or II osteoarthritis can achieve two-year outcomes comparable with those of adolescent and young adult cohorts. However, long-term outcomes in older patients warrant further investigation.

**Take home message**

- Periacetabular osteotomy (PAO) is a safe and effective treatment for carefully selected middle-aged patients with hip dysplasia and Tönnis grade I to II osteoarthritis.

- Although osseous consolidation at the osteotomy sites requires a longer duration in older populations, these patients achieve two-year radiological, functional, and complication outcomes comparable to those of adolescent and young adult cohorts.

## Data Availability

The data that support the findings for this study are available to other researchers from the corresponding author upon reasonable request.

## References

[1] Gala L Clohisy JC Beaulé PE Hip dysplasia in the young adult J Bone Joint Surg Am 2016 98-A 1 63 73 10.2106/JBJS.O.00109 26738905

[2] Aitken HD Westermann RW Bartschat NI et al. Chronically elevated contact stress exposure correlates with intra-articular cartilage degeneration in patients with concurrent acetabular dysplasia and femoroacetabular impingement J Orthop Res 2022 40 11 2632 2645 10.1002/jor.25285 35088436 PMC9325915

[3] Ganz R Blümel S Schleicher A Öttl F Stadelmann VA Leunig M Radiological results of the Bernese periacetabular osteotomy performed before closure of the triradiate cartilage Bone Jt Open 2025 6 6 Supple B 24 32 10.1302/2633-1462.66.BJO-2024-0204.R1 40449897 PMC12184719

[4] Garbuz DS Awwad MA Duncan CP Periacetabular osteotomy and total hip arthroplasty in patients older than 40 years J Arthroplasty 2008 23 7 960 963 10.1016/j.arth.2007.08.015 18534506

[5] Kalore NV Cheppalli SPR Daner WE Jiranek WA Acetabular dysplasia in middle-aged patients: periacetabular osteotomy or total hip arthroplasty? J Arthroplasty 2016 31 9 1894 1898 10.1016/j.arth.2016.02.032 27017199

[6] Sivamurugan G Westermann RW Glass N et al. Incidence and risk factors for non-union of the superior ramus osteotomy when hip dysplasia is treated with periacetabular osteotomy J Hip Preserv Surg 2023 10 2 80 86 10.1093/jhps/hnad006 37900885 PMC10604061

[7] Salih S Groen F Hossein F Witt J Hypermobility, age 40 years or older and BMI >30 kg m^-2^ increase the risk of complications following peri-acetabular osteotomy J Hip Preserv Surg 2020 7 3 511 517 10.1093/jhps/hnaa041 33948206 PMC8081425

[8] Clohisy JC Schutz AL St John L Schoenecker PL Wright RW Periacetabular osteotomy: a systematic literature review Clin Orthop Relat Res 2009 467 8 2041 2052 10.1007/s11999-009-0842-6 19381741 PMC2706361

[9] Tönnis D Heinecke A Acetabular and femoral anteversion: relationship with osteoarthritis of the hip J Bone Joint Surg Am 1999 81-A 12 1747 1770 10.2106/00004623-199912000-00014 10608388

[10] Ganz R Klaue K Vinh TS Mast JW A new periacetabular osteotomy for the treatment of hip dysplasias. Technique and preliminary results Clin Orthop Relat Res 1988 232 232 26 36 3383491

[11] Novais EN Duncan S Nepple J Pashos G Schoenecker PL Clohisy JC Do radiographic parameters of dysplasia improve to normal ranges after bernese periacetabular osteotomy? Clin Orthop Relat Res 2017 475 4 1120 1127 10.1007/s11999-016-5077-8 27646418 PMC5339125

[12] Megerian MF Strony JT Mengers SR Joseph NM Salata MJ Wetzel RJ Use of anatomic radiographic horizons for the lateral center-edge angle in the classification of hip dysplasia Am J Sports Med 2022 50 13 3610 3616 10.1177/03635465221125784 36220151

[13] Sharp IK Acetabular dysplasia: the acetabular angle J Bone Joint Surg Br 1961 43-B 2 268 272 10.1302/0301-620X.43B2.268

[14] Delaunay S Dussault RG Kaplan PA Alford BA Radiographic measurements of dysplastic adult hips Skeletal Radiol 1997 26 2 75 81 10.1007/s002560050197 9060097

[15] Huskisson EC Measurement of pain Lancet 1974 2 7889 1127 1131 10.1016/s0140-6736(74)90884-8 4139420

[16] Harris WH Traumatic arthritis of the hip after dislocation and acetabular fractures: treatment by mold arthroplasty. An end-result study using a new method of result evaluation J Bone Joint Surg Am 1969 51-A 4 737 755 5783851

[17] Byrd JW Jones KS Prospective analysis of hip arthroscopy with 2-year follow-up Arthroscopy 2000 16 6 578 587 10.1053/jars.2000.7683 10976117

[18] Kinoshita K Fujita J Seo H et al. Should discontinuity of the osteotomy site 1 year after periacetabular osteotomy be diagnosed as delayed union and not non-union? J Orthop Sci 2025 30 4 633 639 10.1016/j.jos.2024.10.002 39547853

[19] Selberg CM Davila-Parrilla AD Williams KA Kim Y-J Millis MB Novais EN What proportion of patients undergoing bernese periacetabular osteotomy experience nonunion, and what factors are associated with nonunion? Clin Orthop Relat Res 2020 478 7 1648 1656 10.1097/CORR.0000000000001296 32452931 PMC7310515

[20] Beyer RSH Goodspeed DC Call CJ Mosiman SJ Spiker AM Risk factors associated with nonunion following periacetabular osteotomy for treatment of symptomatic hip dysplasia JB JS Open Access 2025 10 4 e25.00181 10.2106/JBJS.OA.25.00181 41210902 PMC12594298

[21] Ficat RP Idiopathic bone necrosis of the femoral head. Early diagnosis and treatment J Bone Joint Surg Br 1985 67-B 1 3 9 10.1302/0301-620X.67B1.3155745 3155745

[22] Teratani T Naito M Kiyama T Maeyama A Periacetabular osteotomy in patients fifty years of age or older: surgical technique J Bone Joint Surg Am 2011 93-A Suppl 1 30 39 10.2106/JBJS.J.01126 21411684

[23] Yilmaz M Aydin M Capkin S Acetabular dysplasia: a comparison of periacetabular osteotomy results of patients older and younger than 35 years Eur Rev Med Pharmacol Sci 2022 26 22 8303 8310 10.26355/eurrev_202211_30362 36459013

[24] Hartig-Andreasen C Troelsen A Thillemann TM Søballe K What factors predict failure 4 to 12 years after periacetabular osteotomy? Clin Orthop Relat Res 2012 470 11 2978 2987 10.1007/s11999-012-2386-4 22576934 PMC3462869

[25] Dornacher D Lutz B Fuchs M Zippelius T Reichel H Treatment of borderline hip dysplasia with triple pelvic osteotomy: preoperative values of acetabular index and lateral center edge angle can indicate overcorrection Arch Orthop Trauma Surg 2023 143 10 6139 6146 10.1007/s00402-023-04920-z 37272987 PMC10491512

[26] Kitamura K Fujii M Motomura G et al. A computer modeling-based target zone for transposition osteotomy of the acetabulum in patients with hip dysplasia J Bone Joint Surg Am 2024 106-A 24 2347 2355 10.2106/JBJS.23.01132 39418339

[27] Muffly BT Zacharias AJ Jochimsen KN et al. Age at the time of surgery is not predictive of early patient-reported outcomes after periacetabular osteotomy J Arthroplasty 2021 36 10 3388 3391 10.1016/j.arth.2021.05.029 34120795

[28] Ito H Tanino H Yamanaka Y Minami A Matsuno T Intermediate to long-term results of periacetabular osteotomy in patients younger and older than forty years of age J Bone Joint Surg Am 2011 93-A 14 1347 1354 10.2106/JBJS.J.01059 21792502

[29] Yamate S Hamai S Lyman S Kitamura K Motomura G Nakashima Y Age at periacetabular osteotomy does not reduce the likelihood of excellent 20-year patient-reported outcomes in native hips: a k-means clustering analysis J Arthroplasty 2026 41 3 659 667 10.1016/j.arth.2025.11.006 41213413

[30] Leopold VJ Hipfl C Perka C Hardt S Becker L Periacetabular osteotomy for symptomatic hip dysplasia in middle aged patients: does age alone matter? Arch Orthop Trauma Surg 2024 144 3 1065 1070 10.1007/s00402-023-05160-x 38133805 PMC10896936

[31] Edelstein AI Nepple JJ Abu-Amer W Pascual-Garrido C Goss CW Clohisy JC What mid-term patient-reported outcome measure scores, reoperations, and complications are associated with concurrent hip arthroscopy and periacetabular osteotomy to treat dysplasia with associated intraarticular abnormalities? Clin Orthop Relat Res 2021 479 5 1068 1077 10.1097/CORR.0000000000001599 33300755 PMC8051986

[32] Okoroafor UC Pascual-Garrido C Schwabe MT Nepple JJ Schoenecker PL Clohisy JC Activity level maintenance at midterm follow-up among active patients undergoing periacetabular osteotomy Am J Sports Med 2019 47 14 3455 3459 10.1177/0363546519881421 31689124

[33] Wells J Schoenecker P Duncan S Goss CW Thomason K Clohisy JC Intermediate-term hip survivorship and patient-reported outcomes of periacetabular osteotomy: the Washington University Experience J Bone Joint Surg Am 2018 100-A 3 218 225 10.2106/JBJS.17.00337 29406343

[34] Berzolla E Chen L Gosnell GG et al. Defining clinically important outcomes for the modified Harris Hip Score and nonarthritic hip scope for hip arthroscopy to treat femoroacetabular impingement at a minimum 10-year follow-up Am J Sports Med 2025 53 9 2217 2222 10.1177/03635465251344594 40516096

[35] Tønning LU O’Brien M Semciw A Stewart C Kemp JL Mechlenburg I Periacetabular osteotomy to treat hip dysplasia: a systematic review of harms and benefits Arch Orthop Trauma Surg 2023 143 6 3637 3648 10.1007/s00402-022-04627-7 36175675

[36] Sabbag CM Nepple JJ Pascual-Garrido C Lalchandani GR Clohisy JC Sierra RJ The addition of hip arthroscopy to periacetabular osteotomy does not increase complication rates: a prospective case series Am J Sports Med 2019 47 3 543 551 10.1177/0363546518820528 30730756

[37] Tai T-W Gonzalez-Bravo AE Guarin Perez SF Restrepo DJ Sierra RJ How to minimize complications during and after periacetabular osteotomy: lessons learned from over 700 patients with and without concomitant hip arthroscopy Bone Jt Open 2025 6 10 2022 2031 10.1302/2633-1462.610.BJO-2025-0194 41062091 PMC12508929

[38] Matsunaga A Akiho S Kinoshita K Naito M Yamamoto T The prevalence and risk factors for delayed union of the superior pubic ramus at one year after curved periacetabular osteotomy: its risk factor and outcome Int Orthop 2018 42 6 1253 1258 10.1007/s00264-017-3706-9 29209742

[39] Sharma S Shah R Draviraj KP Bhamra MS Use of telephone interviews to follow up patients after total hip replacement J Telemed Telecare 2005 11 4 211 214 10.1258/1357633054068883 15969797

